# Assembly of a series of zinc coordination polymers based on 5-functionalized isophthalic acids and dipyridyl†

**DOI:** 10.1039/c7ra12874f

**Published:** 2018-02-16

**Authors:** Tao Wang, Rong-Rong Zhu, Xiao-Feng Zhang, Tong Yan, Quan Wang, Jing Feng, Jie Zhou, Lin Du, Qi-Hua Zhao

**Affiliations:** Key Laboratory of Medicinal Chemistry for Natural Resource Education Ministry, School of Chemical Science and Technology – Pharmacy, Yunnan University Kunming 650091 PR China qhzhao@ynu.edu.cn lindu@ynu.edu.cn

## Abstract

To investigate the influence of different 5-functionalized isophthalate ligands on the self-assembly and structures of Zn(ii)-based coordination polymers (CPs), we selected four 5-functionalized isophthalate ligands coordinated with 4,4′-bipyridyl (bpy) and Zn(ii) salt, and four new CPs, namely, {[Zn(EtOip)(bpy)]·2H_2_O}_*n*_ (1), {[Zn(PrOip)(bpy)]·2H_2_O}_*n*_ (2), [Zn(^*n*^BuOip)(bpy)]_*n*_ (3) and [Zn(^*n*^PeOip)(bpy)]_*n*_ (4) (where EtOip = 5-ethoxyisophthalate, PrOip = 5-*n*-propoxyisophthalate, ^*n*^BuOip = 5-*n*-butoxyisophthalate and ^*n*^PeOip = 5-*n*-pentyloxyisophthalate), were prepared under hydrothermal conditions. Moreover, we considered changing the features of the N-ligands (length), which are expected to alter the structural diversities and properties of the resultant CPs. We used 1,2-bis(4-pyridyl)ethene (bpe) to replace bpy; and obtained four new coordination polymers (CPs), namely, {[Zn(EtOip)(bpe)]·H_2_O}_*n*_ (5), {[Zn(PrOip)(bpe)]·H_2_O}_*n*_ (6), {[Zn(^*n*^BuOip)(bpe)]·0.5DMF}_*n*_ (7) and [Zn(^*n*^PeOip)(bpe)]_*n*_ (8). All of these compounds were characterized by single crystal X-ray diffraction, infrared spectroscopy, elemental analysis and powder X-ray diffraction measurements. Single-crystal X-ray analysis reveals that the structure of complexes 1–4 are very similar, although different functionalized-isophthalate ligands are employed. Complexes 5, 6 and 7 possess a two-dimensional (2D) layered structure, and the 2D framework of complexes 5 and 7 can be rationalized to be a threefold interpenetrating four-connected {4^4^·6^2^} topological **sql** network, while 6 exhibits a twofold interpenetrating 4-connected 2D framework. Compound 8 features a 6-connected 3D topology net based on a novel bimetallic unit [(Zn)_2_(CO_2_)_2_] linked by mixed ^*n*^PeOip and bpe ligands. Moreover, thermal and photoluminescence properties of the synthesized complexes were also studied in detail.

## Abstract 2

A series of zinc coordination polymers based on 5-functionalized isophthalate and flexible dipyridyl ligands have been prepared. Their thermal and luminescent properties in the solid state were also investigated.

## Graphical abstract

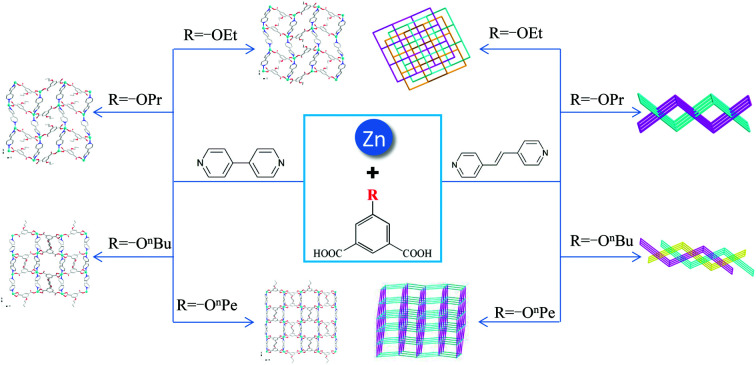

## Introduction

Over the past few decades, the rational design and synthesis of novel coordination polymers (CPs) has been of great interest in coordination chemistry and material science due to the intriguing architectures and topologies of the CPs, but also for their potential applications as functional materials in numerous fields, such as luminescence, catalysis, gas adsorption/separation, magnetism and so forth.^1–13^ Despite that a comparatively large number of interesting coordination polymers with intricate structural architectures have been reported to date, the design and construction of multi-functional CPs with desired structures and properties remains a significant challenge for chemists. This is because many intrinsic and external factors, such as the coordination nature of the metal centers, the structural characteristics of the organic ligands, stoichiometry, temperature, solvent, the pH of the solution and so on, may considerably influence the formation of the resulting CPs.^14–22^ Of all the influencing factors, the deliberate selection of functionalized organic ligands plays a crucially important role in the structural assembly process, and in some cases, a subtle alteration of organic motifs may lead to a novel architecture.^23–25^

In recent years, functionalized polycarboxylic acid derivatives have been extensively used as multifunctional organic ligands in the formation of coordination frameworks owing to their high stability, various coordination modes and strong coordination ability toward transition metal ions.^26–30^ Among them, 5-functionalized isophthalates are good candidates within coordination and supramolecular chemistry because their two V-shaped carboxylate groups may bridge metal ions or metal clusters *via* versatile coordination modes similar to that of isophthalate.^31–40^ For example, McCormick *et al.* found that the topology of CPs is dependent on the combination of solvent system used and length of the functionalized-groups.^39^ The studies of Sha *et al.* indicated that the adsorption capacity of water, methanol and ethanol can be effectively tuned through changing the functionalized-groups.^40^ Up to now, a large number of CPs based on 5-functionalized isophthalate derivatives have been reported by several groups. However, studies on the effect of long alkoxy chain functionalized-groups on the phenolic oxygen atom in the formation of the resultant CPs remain scarce so far.

In addition, the introduction of N-containing ligands into the carboxylate systems may exert a significant influence on the assembly process of CPs. Previous studies have demonstrated that N-donor auxiliary ligands can provide cooperative coordination together with the carboxylate group to satisfy the requirements of the coordination geometries of the metal ions in the assembly process. Bipyridyl ligands, such as 4,4′-bipyridyl (bpy)^41–50^ and 1,2-bis(4-pyridyl)ethene (bpe)^41–43,51–61^ have been extensively used as ancillary ligands in the controllable synthesis of CPs.

Inspired from the above-mentioned, we therefore selected four 5-functionalized isophthalate ligands with different numbers of carbon atoms, which are 5-ethoxyisophthalate (EtOip), 5-*n*-propoxyisophthalate (PrOip), 5-*n*-butoxyisophthalate (^*n*^BuOip) and 5-*n*-pentyloxyisophthalate (^*n*^PeOip) and neutral N-donor ligands 4,4′-bipyridine (bpy) and 1,2-bis(4-pyridyl)ethane (bpe) as an organic building blocks. Eight new Zn CPs, namely, {[Zn(EtOip)(bpy)]·2H_2_O}_*n*_ (1), {[Zn(PrOip)(bpy)]·2H_2_O}_*n*_ (2), [Zn(^*n*^BuOip)(bpy)]_*n*_ (3), [Zn(^*n*^PeOip)(bpy)]_*n*_ (4), {[Zn(EtOip)(bpe)]·H_2_O}_*n*_ (5), {[Zn(PrOip)(bpe)]·H_2_O}_*n*_ (6), {[Zn(^*n*^BuOip)(bpe)]·0.5DMF}_*n*_ (7) and [Zn(^*n*^PeOip)(bpe)]_*n*_ (8), have been synthesized under hydrothermal conditions. Their structural diversities reveal that different functionalized groups and the length of dipyridyl ligands play an important role in the self-assembly processes. All compounds were characterized by single-crystal X-ray diffraction, infrared spectra (IR), elemental analyses and powder X-ray diffraction (PXRD). Furthermore, thermal and photoluminescence properties of the synthesized complexes were also studied in detail.

## Experimental

### Materials and methods

The ligands H_2_EtOip, H_2_PrOip, H_2_^*n*^BuOip and H_2_^*n*^PeOip were synthesized according to a method in the literature.^39,62^ Other reagents and solvents were commercially available and were used without further purification. Elemental analyses for C, H, and N were carried out using an Elementar Vario ELIII analyzer. IR spectra were recorded on a FT-IR Thermo Nicolet Avatar 360 using KBr pellets in the 400–4000 cm^−1^ region. All powder X-ray diffraction (PXRD) analyses were recorded on a Rigaku D/M-2200T automated diffractometer (CuKα, 1.5418 Å). Thermal stability studies were carried out on a NETZSCH STA-449C thermoanalyzer with a heating rate of 10 °C min^−1^ under a nitrogen atmosphere. All fluorescence measurements were performed on an Edinburgh Instrument F920 spectrometer.

### Synthesis procedures

#### Preparation of {[Zn(EtOip)(bpy)]·2H_2_O}_*n*_ (1)

A mixture of H_2_EtOip (21 mg, 0.1 mmol), bpy (15.6 mg, 0.1 mmol), Zn(NO_3_)_2_·6H_2_O (30 mg, 0.1 mmol) dissolved in DMF (1.0 mL) and H_2_O (4.0 mL) were placed in a 15 mL Teflon-lined stainless steel autoclave and heated at 120 °C for two days. After the mixture was cooled to ambient temperature at a rate of 5 °C h^−1^, pink block crystals of 1 were obtained with a yield of 46% (based on Zn). Anal. calcd for C_20_H_20_N_2_O_7_Zn: C, 51.57%; H, 4.33%; N, 6.02%. Found: C, 51.63%; H, 4.41%; N, 6.06%. IR (KBr, cm^−1^): 3474(vs), 2932(w), 2868(w), 1620(s), 1589(w), 1506(w), 1409(s), 1347(w), 1261(w), 1227(w), 1122(m), 1068(w), 1028(w), 929(w), 844(w), 779(w), 731(m).

#### Preparation of {[Zn(PrOip)(bpy)]·2H_2_O}_*n*_ (2)

The procedures for the syntheses of 2 were similar to that used for 1, except that H_2_PrOip (22 mg, 0.1 mmol) was used instead of H_2_EtOip. Pink block crystals of 2 were obtained with a yield of 56% (based on Zn). Anal. calcd for C_21_H_22_N_2_O_7_Zn: C, 52.57%; H, 4.62%; N, 5.84%. Found: C, 52.46%; H, 4.57%; N, 5.68%. IR (KBr, cm^−1^): 3417(vs), 2967(w), 1618(s), 1574(w), 1491(w), 1418(s), 1320(w), 1264(w), 1220(w), 1121(m), 1089(w), 1013(w), 914(w), 875(w), 777(w), 729(m), 641(w).

#### Preparation of [Zn(^*n*^BuOip)(bpy)]_*n*_ (3)

The procedures for the syntheses of 3 were similar to that used for 1, except that H_2_^*n*^BuOip (22 mg, 0.1 mmol) was used instead of H_2_EtOip. Colourless block crystals of 3 were obtained with a yield of 60% (based on Zn). Anal. calcd for C_22_H_20_N_2_O_5_Zn: C, 57.72%; H, 4.40%; N, 6.12%. Found: C, 57.67%; H, 4.36%; N, 6.15%. IR (KBr, cm^−1^): 3414(vs), 2957(w), 2870(w), 1611(s), 1549(w), 1487(w), 1420(s), 1320(w), 1268(w), 1220(w), 1123(m), 1099(w), 1015(w), 992(w), 854(w), 781(w), 736(m), 678(w), 637(w).

#### Preparation of [Zn(^*n*^PeOip)(bpy)]_*n*_ (4)

The procedures for the syntheses of 4 were similar to that used for 1, except that H_2_^*n*^PeOip (22 mg, 0.1 mmol) was used instead of H_2_EtOip. Colourless block crystals of 8 were obtained with a yield of 54% (based on Zn). Anal. calcd for C_23_H_22_N_2_O_5_Zn: C, 58.55%; H, 4.70%; N, 5.94%. Found: C, 58.57%; H, 4.63%; N, 5.86%. IR (KBr, cm^−1^): 3438(vs), 2923(w), 2857(w), 1622(w), 1611(s), 1568(w), 1488(w), 1391(s), 1321(w), 1263(w), 1223(w), 1118(m), 1078(w), 1017(w), 824(w), 781(w), 639(w).

#### Preparation of {[Zn(EtOip)(bpe)]·H_2_O}_*n*_ (5)

Compound 5 was prepared by using a method similar to that used for the preparation of 1, except that bpe (18 mg, 0.1 mmol) were used instead of bpy. Colourless block crystals of 5 were obtained with a yield of 59% (based on Zn). Anal. calcd for C_22_H_20_N_2_O_6_Zn: C, 55.77%; H, 4.26%; N, 5.91%. Found: C, 55.69%; H, 4.18%; N, 5.86%. IR (KBr, cm^−1^): 3516(vs), 2924(w), 2880(w), 1615(s), 1580(w), 1508(w), 1477(s), 1435, 1342(w), 1262(w), 1207(w), 1118(m), 1031(w), 936(w), 777(w), 733(m).

#### Preparation of {[Zn(PrOip)(bpe)]·H_2_O}_*n*_ (6)

Compound 6 was prepared by using a method similar to that used for the preparation of 2, except that bpe (18 mg, 0.1 mmol) were used instead of bpy. Colourless block crystals of 6 were obtained with a yield of 52% (based on Zn). Anal. calcd for C_23_H_22_N_2_O_6_Zn: C, 56.63%; H, 4.55%; N, 5.74%. Found: C, 56.65%; H, 4.53%; N, 5.68%. IR (KBr, cm^−1^): 3466(vs), 2959(w), 2878(w), 1613(s), 1587(w), 1450(w), 1404(s), 1337(w), 1262(w), 1203(w), 1121(m), 1026(w), 963(w), 843(w), 777(w), 730(m).

#### Preparation of {[Zn(^*n*^BuOip)(bpe)]·0.5DMF}_*n*_ (7)

Compound 7 was prepared by using a method similar to that used for the preparation of 3, except that bpe (18 mg, 0.1 mmol) were used instead of bpy. Colourless block crystals of 7 were obtained with a yield of 36% (based on Zn). Anal. calcd for C_24_H_22_N_2_O_5_Zn: C, 59.58%; H, 4.58%; N, 5.79%. Found: C, 59.53%; H, 4.62%; N, 5.76%. IR (KBr, cm^−1^): 3439(vs), 2933(w), 2868(w), 1679(s), 1613(s), 1578(w), 1474(w), 1434(s), 1344(w), 1262(w), 1206(w), 1119(w), 1069(w), 992(w), 835(w), 780(w), 732(m), 694(w), 621(w).

#### Preparation of [Zn(^*n*^PeOip)(bpe)]_*n*_ (8)

Compound 8 was prepared by using a method similar to that used for the preparation of 4, except that bpe (18 mg, 0.1 mmol) were used instead of bpy. Colourless block crystals of 8 were obtained with a yield of 61% (based on Zn). Anal. calcd for C_19_H_19_NO_5_Zn: C, 56.10%; H, 4.71%; N, 3.44%. Found: C, 56.08%; H, 4.65%; N, 3.34%. IR (KBr, cm^−1^): 3446(vs), 2953(w), 2868(w), 1614(s), 1563(w), 1453(w), 1358(s), 1320(w), 1266(w), 1209(w), 1123(m), 1104(w), 1044(w), 841(w), 781(w), 726(m).

### X-ray data collection and structure determination

Single-crystal X-ray diffraction data for 1–8 were collected on a Bruker SMART APEX II CCD diffractometer equipped with graphite-monochromated Mo-Kα (*λ* = 0.71073 Å) by using the *Φ*/*ω* scan technique. Absorption correction was based on symmetry equivalent reflections by using the SADABS program.^63^ The crystal structures of 1–8 were solved by direct methods and refined on *F*^2^ by full-matrix least-squares methods with the SHELXL-2014 program.^64^ All non-hydrogen atoms were refined anisotropically. The H atoms of the solvent water molecule in 1, 2, 5 and 6 were located from the Fourier map and included in the final refinement by use of geometrical restraints with the O–H distances being fixed at 0.85 Å and *U*_iso_(H) equivalent to 1.5 times of *U*_eq_(O). All of the other H atoms were introduced at the calculated positions and included in the structure-factor calculations. The highly disordered DMF molecules in 7 were removed using the SQUEEZE procedure in PLATON.^65^ The number of DMF molecules was obtained on the basis of elemental and thermogravimetric analyses. The disordered C and O atoms of the EtOip ligands in compound 1 (C19, C19′, C20, C20′, O5, O5′), the disordered C and O atoms of the PrOip ligands in compound 2 (C19, C19′, C20, C20′, C21, C21′, O5, O5′), the disordered C atoms of the bpy ligands in compound 4 (C1, C1′, C2, C2′, C4, C4′, C5, C5′) and the disordered O atoms of the water molecules in compound 1 (O2W, O2W′) were refined using O atom split over two sites, with a total occupancy of 1. A summary of key crystallographic information for 1–8 is given in Table 1. Selected bond lengths and angles for 1–8 are listed in Table S1.†

**Table 1:** Crystallographic data and structure refinement summary for compounds 1–8

Compound	1	2	3	4	5	6	7	8
Formula	C_20_H_20_N_2_O_7_Zn	C_21_H_22_N_2_O_7_Zn	C_22_H_20_N_2_O_5_Zn	C_23_H_22_N_2_O_5_Zn	C_22_H_20_N_2_O_6_Zn	C_23_H_22_N_2_O_6_Zn	C_24_H_22_N_2_O_5_Zn	C_19_H_19_NO_5_Zn
Formula weight	465.75	479.77	457.77	471.81	473.77	487.79	483.80	406.72
Crystal system	Monoclinic	Monoclinic	Orthorhombic	Orthorhombic	Monoclinic	Triclinic	Triclinic	Monoclinic
Space group	*P*2_1_/*n*	*P*2_1_/*n*	*Pbca*	*Cmce*	*P*2_1_/*c*	*P*1̄	*P*1̄	*C*2/*c*
*a* (Å)	7.9207(7)	7.9431(7)	13.5909(16)	18.696(2)	8.6150(15)	9.5648(16)	8.482(7)	16.6370(16)
*b* (Å)	15.0598(13)	15.5159(14)	15.3382(18)	14.2482(17)	26.006(4)	11.4815(16)	10.245(8)	9.2945(9)
*c* (Å)	17.2160(14)	17.2037(16)	19.411(2)	16.1635(19)	10.0175(17)	11.5626(16)	15.967(12)	24.212(2)
*α* (°)	90	90	90	90	90	100.477(2)	99.022(10)	90
*β* (°)	99.0770(10)	99.5690(10)	90	90	113.667(2)	100.808(2)	93.005(10)	94.950(1)
*γ* (°)	90	90	90	90	90	113.570(2)	114.363(9)	90
*V* (Å^3^)	2027.9(3)	2090.8(3)	4046.4(8)	4305.7(9)	2055.6(6)	1095.7(3)	1237.7(17)	3730.0(6)
*Z*	4	4	8	8	4	2	2	8
*D*_calcd_ (g cm^−3^)	1.526	1.524	1.503	1.456	1.531	1.479	1.298	1.449
*μ* (mm^−1^)	1.256	1.221	1.251	1.178	1.24	1.16	1.03	1.35
*F* (000)	960.0	992.0	1888.0	1952.0	976	504	500	1680
*R*_1_ [*I* > 2*σ*(*I*)]	0.0329	0.0338	0.0457	0.0704	0.0487	0.0399	0.0396	0.0321
w*R*_2_ [*I* > 2*σ*(*I*)]	0.0876	0.0809	0.0914	0.1897	0.0975	0.0825	0.0851	0.0809
*R*_1_ (all data)	0.0401	0.0460	0.1146	0.0950	0.0980	0.0618	0.0543	0.0403
w*R*_2_ (all data)	0.0913	0.0878	0.1153	0.2004	0.1112	0.0896	0.0905	0.0848
GOF on *F*^2^	1.10	1.03	0.99	1.09	0.98	1.02	0.97	1.07
CCDC number	1557106	1557107	1557108	1557109	1557102	1557103	1557104	1557105

## Results and discussion

### Crystal structures of 1 and 2

**Fig. 1:**
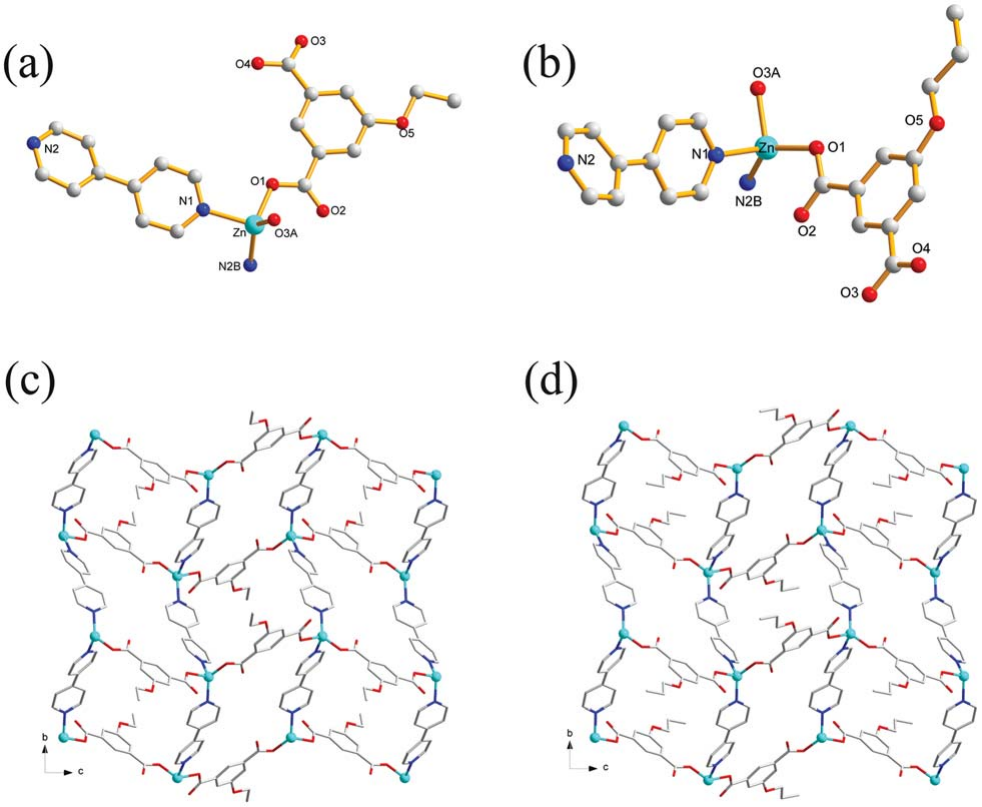
(a) Coordination environment of the Zn(ii) centers in 1 with the hydrogen atoms and solvent molecules omitted for clarity (symmetric codes: (A) *x* − 1/2, −*y* + 3/2, *z* − 1/2 and (B) −*x* − 1/2, *y* + 1/2, −*z* + 1/2). (b) Coordination environment of the Zn(ii) centers in 2 with the hydrogen atoms and solvent molecules omitted for clarity (symmetric codes: (A) *x* + 1/2, −*y* + 3/2, *z* + 1/2 and (B) −*x* + 1/2, *y* + 1/2, −*z* + 1/2). (c) View of the 2D [Zn(EtOip)(bpy)]_*n*_ network of 1. (d) View of the 2D [Zn(PrOip)(bpy)]_*n*_ network of 2.

**Scheme 1:**
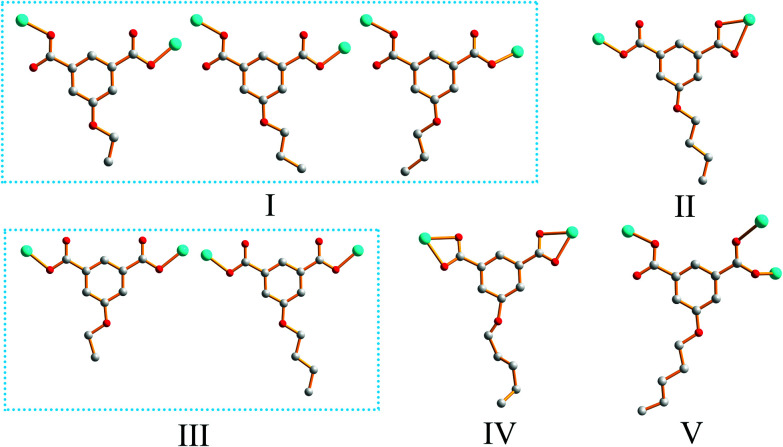
Coordination modes of the 5-functionalized isophthalate ligands used in this work.

**Fig. 2:**
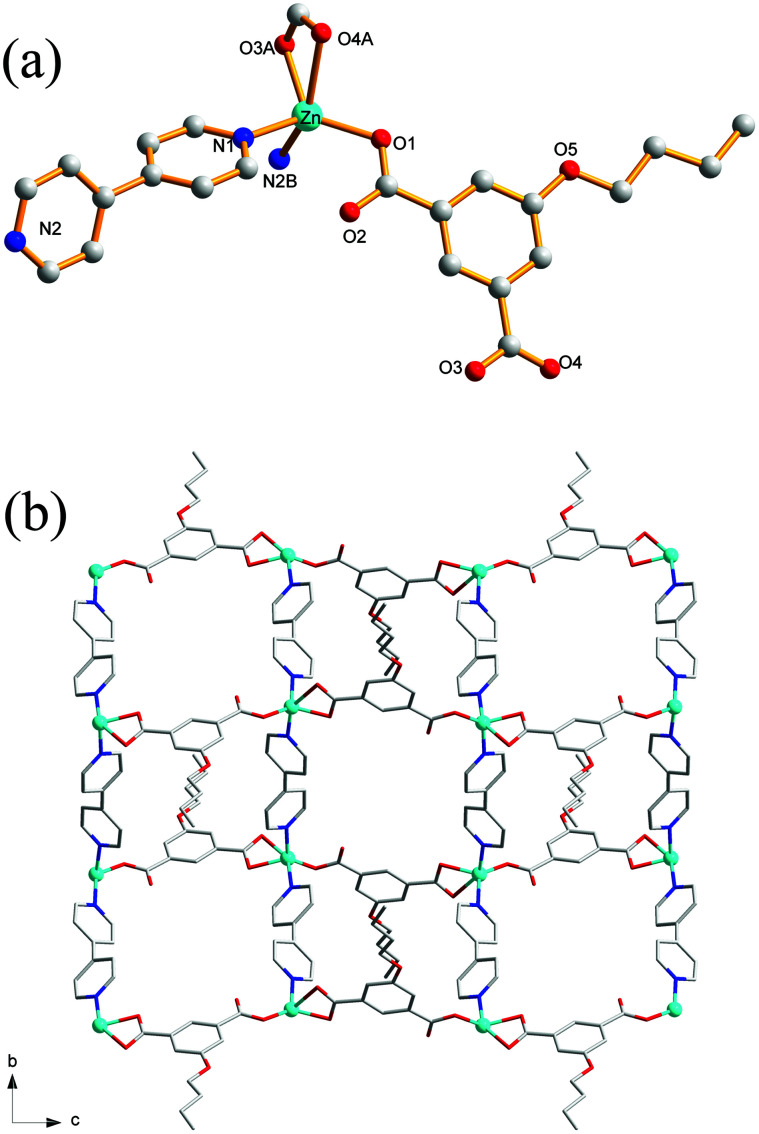
(a) Coordination environment of the Zn(ii) centers in 3 with the hydrogen atoms omitted for clarity (symmetric codes: (A) *x*, −*y* + 1/2, *z* + 1/2 and (B) −*x* + 1, *y* − 1/2, −*z* + 1/2). (b) View of the 2D [Zn(^*n*^BuOip)(bpy)]_*n*_ network of 3.

Single-crystal XRD analysis revealed that the structures of 1 and 2 are highly similar, and thus can be treated together. The asymmetric units of 1 (Fig. 1a) and 2 (Fig. 1b) contain one Zn(ii) ion, one bpy ligand, two water solvent molecules and one EtOip ligand for 1 or one PrOip ligand for 2. Each Zn center is coordinated by two nitrogen atoms from two different bpy ligands and two oxygen atoms from two different EtOip ligands for 1 or PrOip ligands for 2. The Zn atoms are connected by bpy ligands to form an infinite 1D zigzag [Zn(bpy)]_*n*_ chain running along the *b* axis (Fig. S1†). Each 1D chain further bridges its equivalent ones *via* EtOip (1) or PrOip (2) along the *c* axis and finally affords a 2D coordination polymer network (Fig. 1c and d). In 1 and 2, both EtOip and PrOip molecule adopt a bis-monodentate coordination mode (Scheme 1(I)). The Zn⋯Zn distance bridged by the 5-functionalized isophthalate linker is 9.3887(8) Å for 1 and 9.4095(8) Å for 2, whereas the Zn⋯Zn distance bridged by bpy ranges from 10.9766(8) Å to 10.9512(8) Å. Topological analysis reveals that both frameworks can be represented as a uninodal 4-connected **sql**/Shubnikov tetragonal plane net with the point symbol {4^4^·6^2^}.

### Crystal structure of 3

Crystal structure determination reveals that complex 3 crystallizes in the orthorhombic space group *Pbca* and its asymmetric unit bears one Zn(ii) ion, one bpy ligand and one ^*n*^BuOip ligand. As shown in Fig. 2a, the Zn center is five-coordinated with three carboxylate oxygen atoms from two ^*n*^BuOip ligands and two nitrogen atoms derived from two different bpy ligands, thereby resulting a distorted trigonal bipyramidal coordination [ZnN_2_O_3_] geometry. The lengths of the Zn–O [1.935(2)–2.037(2) Å] and Zn–N [2.067(3)–2.120(3) Å] bonds are comparable with those observed in other related compounds containing O–Zn–N segments. Each ^*n*^BuOip ligand in 3 bridges two Zn centres *via* chelating and monodentate carboxylate groups (Scheme 1(II)) to generate a 1D [Zn^*n*^BuOip]_*n*_ chain along the *c* axis (Fig. S2†). The adjacent [Zn^*n*^BuOip]_*n*_ chains are linked by bpy to yield a 2D wrinkled layer of [Zn(^*n*^BuOip)(bpy)]_*n*_ extending in the *bc* plane (Fig. 2b). Topological analysis reveals that the network can also be viewed as a 4-connected **sql**/Shubnikov tetragonal plane net with the point symbol {4^4^·6^2^}.

### Crystal structure of 4

**Fig. 3:**
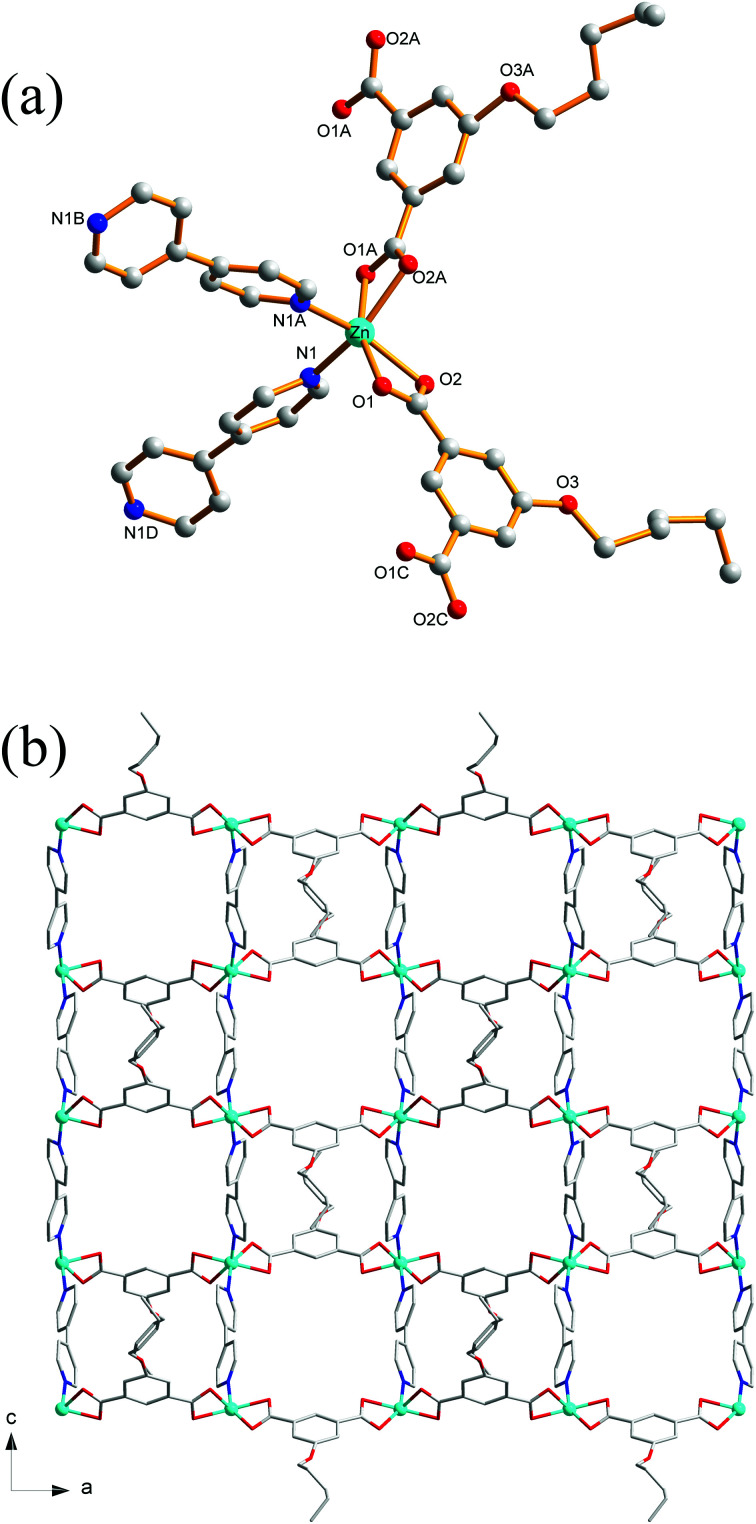
(a) Coordination environment of the Zn(ii) centers in 4 with the hydrogen atoms omitted for clarity (symmetric codes: (A) −*x* + 1/2, *y*, −*z* + 1/2; (B) 1/2 − *x*, 1 − *y*, −1/2 + *z*; (C) 1 − *x*, *y*, *z* and (D) *x*, 1 − *y*, 1 − *z*). (b) View of the 2D [Zn(^*n*^PeOip)(bpy)]_*n*_ network of 4.

Compound 4 crystallizes in the orthorhombic with *Cmca* space group. The asymmetric unit of the title compound comprises one Zn(ii) ion, half of a ^*n*^PeOip linker, as well as half of a bpy ligand. As shown in Fig. 3a, the six-coordinate Zn(ii) ion possesses a distorted octahedral [ZnN_2_O_4_] geometry, which is completed by four carboxylate oxygen atoms from two ^*n*^PeOip ligands and two nitrogen atoms coming from two bpy ligands. The Zn–O [2.173(4)–2.192(4) Å] and Zn–N [2.104(4) Å] distances are within reasonable ranges reported for other related Zn(ii) compounds. Each ^*n*^PeOip connects two Zn atoms *via* chelating bis(bidentate) coordination mode (Scheme 1(IV)) to give an infinite 1D chain (Fig. S3†), and each bpy links two Zn atoms to form an infinite 1D zigzag chain. Then, two kinds of 1D chains cross-link together to give a undulating 2D network of 4 (Fig. 3b). The framework 4 can also be represented as a uninodal 4-connected **sql**/Shubnikov net with the point symbol {4^4^·6^2^}.

### Crystal structure of 5

**Fig. 4:**
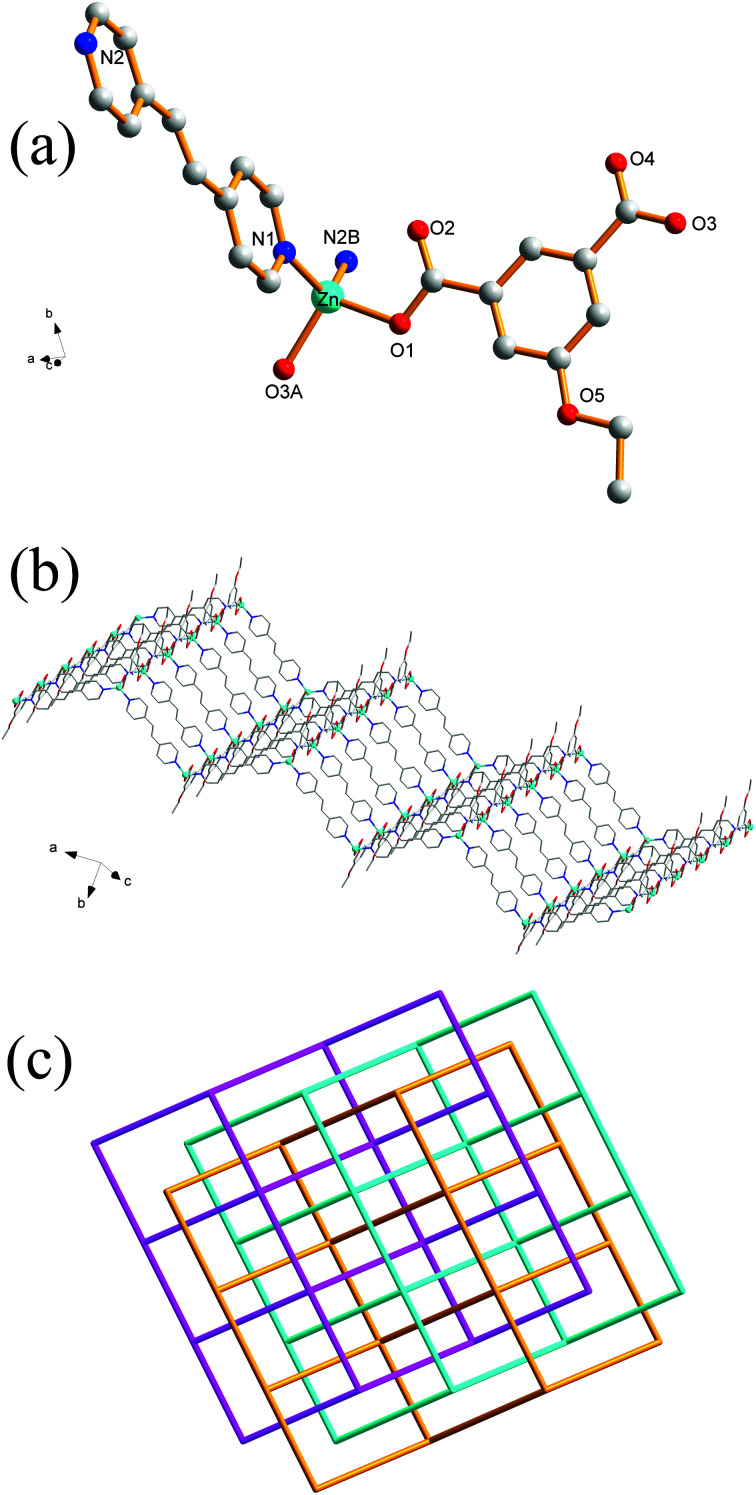
(a) Coordination environment of the Zn(ii) centers in 5 with the hydrogen atoms and solvent molecules omitted for clarity (symmetric codes: (A) *x* + 1, *y*, *z* + 1 and (B) *x* − 1, −*y* + 1/2, *z* + 1/2). (b) View of the 2D [Zn(EtOip)(bpe)]_*n*_ network of 5. (c) The 2D + 2D + 2D → 2D interpenetration network in 5.

Single-crystal X-ray analysis reveals that the complex 5 crystallizes in a monoclinic system with space group *P*2_1_/*c*. There are one Zn(ii) ion, one EtOip, one bpe ligands and one water molecule in the asymmetric unit. As shown in Fig. 4a, the Zn(ii) ion is four-coordinated by two oxygen atoms from different EtOip ligands and two nitrogen atoms from two distinct bpe ligands, thus showing a distorted tetrahedral coordination [ZnN_2_O_2_] geometry. The Zn–O [1.979(3)–1.984(3) Å] and Zn–N [2.030(3) to 2.043(3) Å] bonds are consistent with related literature data. Each Zn(ii) ion bridges two EtOip ligands and two bpe ligands to form a network with large rectangular windows (Fig. 4b). Framework 5 can also be viewed as a 4-connected **sql**/Shubnikov tetragonal plane net with the point symbol {4^4^·6^2^}. Moreover, adjacent separate nets are further interpenetrated with each other, resulting in a threefold 2D interpenetrating tri-layer architecture (Fig. 4c).

### Crystal structure of 6

**Fig. 5:**
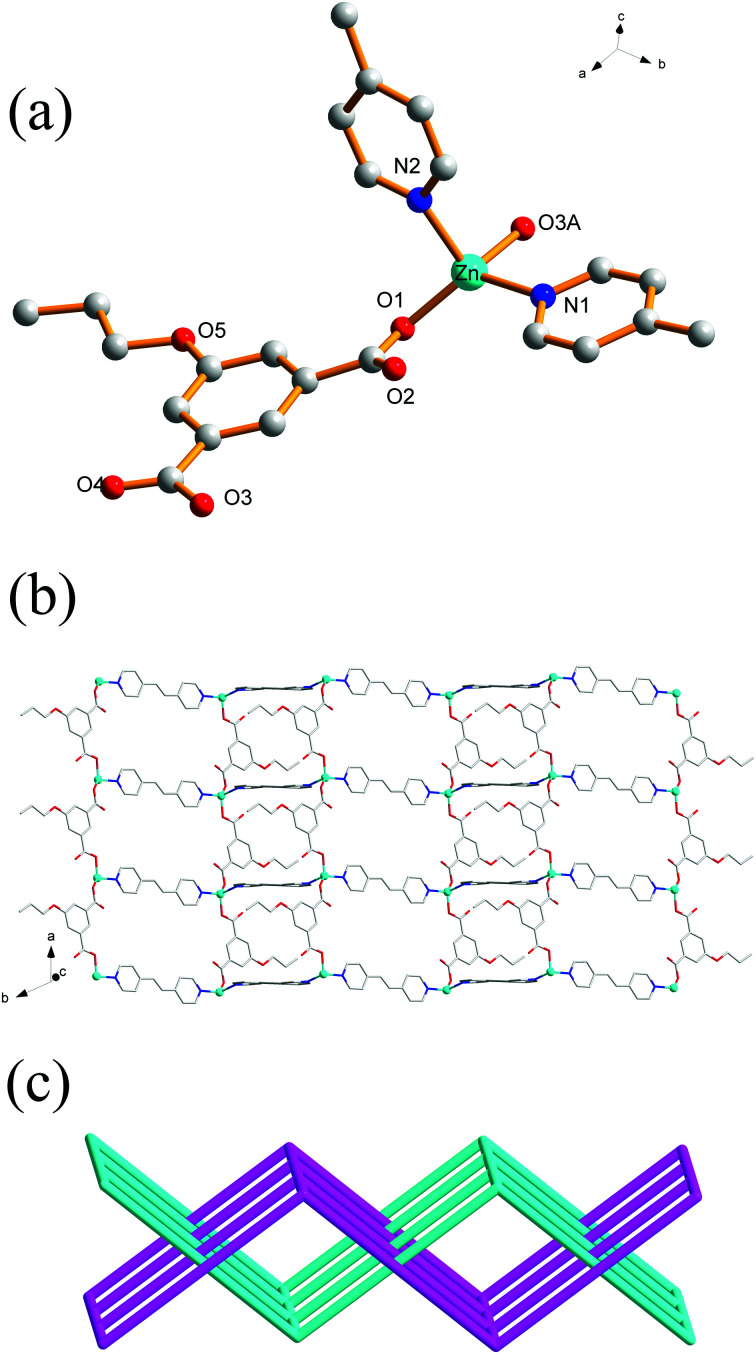
(a) Coordination environment of the Zn(ii) centers in 6 with the hydrogen atoms and solvent molecules omitted for clarity (symmetric codes: (A) *x* − 1, *y*, *z*). (b) View of the 2D [Zn(PrOip)(bpe)]_*n*_ network of 6. (c) The 2D + 2D → 2D interpenetration network in 6.

Different from 5, complex 6 crystallizes in the triclinic crystal system with *P*1̄ space group. The asymmetric unit consists of one Zn(ii) ion, one PrOip ligand and two half of bpe ligands with an inversion centre at the midpoint of the central C=C bond as well as one water molecule. As shown in Fig. 5a, the Zn center is also four-coordinated and features a distorted tetrahedral [ZnO_2_N_2_] geometry, which is bonded by two oxygen atoms originating from different PrOip ligands and two nitrogen atoms belonging to two distinct bpe ligands. The Zn–O [1.9264(19) to 1.939(2) Å] bond distances in 6 are shorter than that of 5. The Zn–N bond length [2.048(2) to 2.068(3) Å] in 6 is slightly longer than that of 5. In 6, a pair of Zn atoms are bridged by PrOip ligands *via* a bis-monodentate coordination mode to form a [Zn(PrOip)]_*n*_ chain along the *c* axis (Scheme 1(I) and Fig. S5†). Such a chain is further connected by bpe ligands to yield a 2D wrinkled layer (Fig. 5b). In addition, the rectangular windows of 6 are filled with another independent net, generating a 2D + 2D → 2D interpenetration bi-layer structure with a {4^4^·6^2^} topology, instead of the threefold interpenetrated framework of 5 (Fig. 5c).

### Crystal structure of 7

**Fig. 6:**
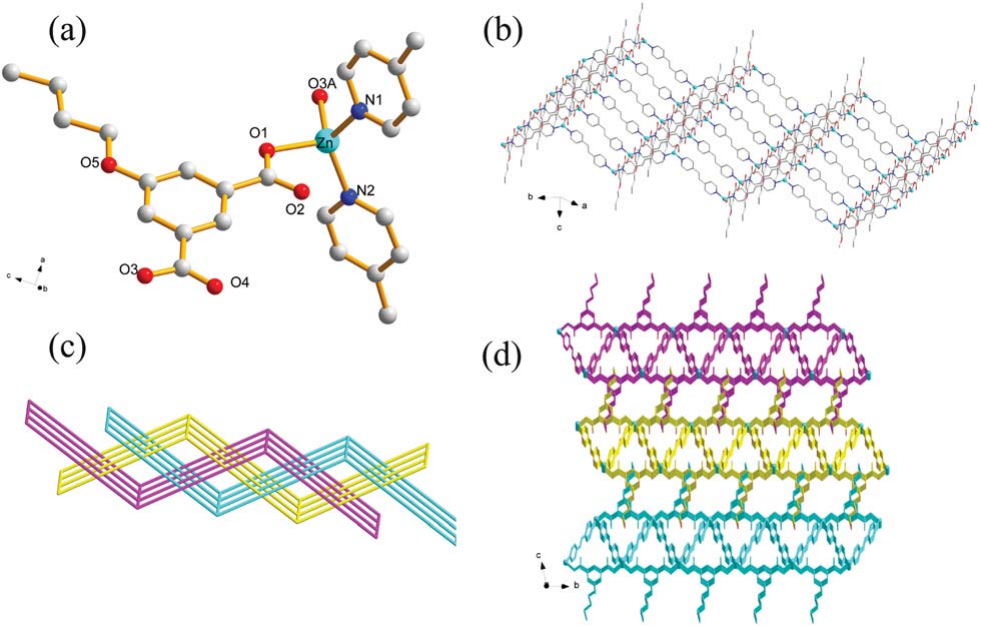
(a) Coordination environment of the Zn(ii) centers in 7 with the hydrogen atoms and solvent molecules omitted for clarity (symmetric codes: (A) *x* + 1, *y* + 1, *z*). (b) View of the 2D [Zn(^*n*^BuOip)(bpe)]_*n*_ network of 7. (c) The 2D + 2D + 2D → 2D interpenetration network in 7. (d) 3D supramolecular structure based on C–H⋯π interaction in 7.

Compound 7 also crystallizes in the triclinic crystal system with *P*1̄ space group. The asymmetric unit of 7 contains one Zn(ii) ion, one ^*n*^BuOip ligand and two half of bpe ligands which has an inversion center at the midpoint of the central C=C bond as well as a half of DMF molecules which confirmed by elemental and thermogravimetric analyses. In 7, the distorted tetrahedral Zn center is coordinated to two oxygen atoms from two ^*n*^BuOip ligands and two nitrogen atoms from two bpe molecules (Fig. 6a). The Zn–O [1.978(2)–1.980(2) Å] and Zn–N [2.035(2)–2.037(2) Å] bond distances of 7 are similar with that of 5. Each ^*n*^BuOip ligand bridges a pair of Zn atoms *via* bis-monodentate coordination mode, to form a 1D [Zn(^*n*^BuOip)]_*n*_ chain extending along the *c* axis (Scheme 1(III) and Fig. S6†). Each bpe ligand links the Zn center in one chain to another Zn center in an adjacent chain, forming a 2D wrinkle-like [Zn(^*n*^BuOip)(bpe)]_*n*_ net with large rectangular windows (Fig. 6b). Furthermore, three adjacent networks interlock with each other to give a threefold interpenetrating architecture with a {4^4^·6^2^} topology (Fig. 6c). It is noted worth that the interpenetrating fashions of 7 is slightly different from 5 due to the structure of 7 containing two types of bpe linkers. Interestingly, adjacent tri-layers in 7 connect with each other through C–H⋯π interactions to form a supramolecular 3D network (Fig. 6d) with a free volume of 18.6% (229.7 Å^3^ out of the 1237.7 Å^3^ unit cell volume) by using PLATON.^66^

### Crystal structure of 8

**Fig. 7:**
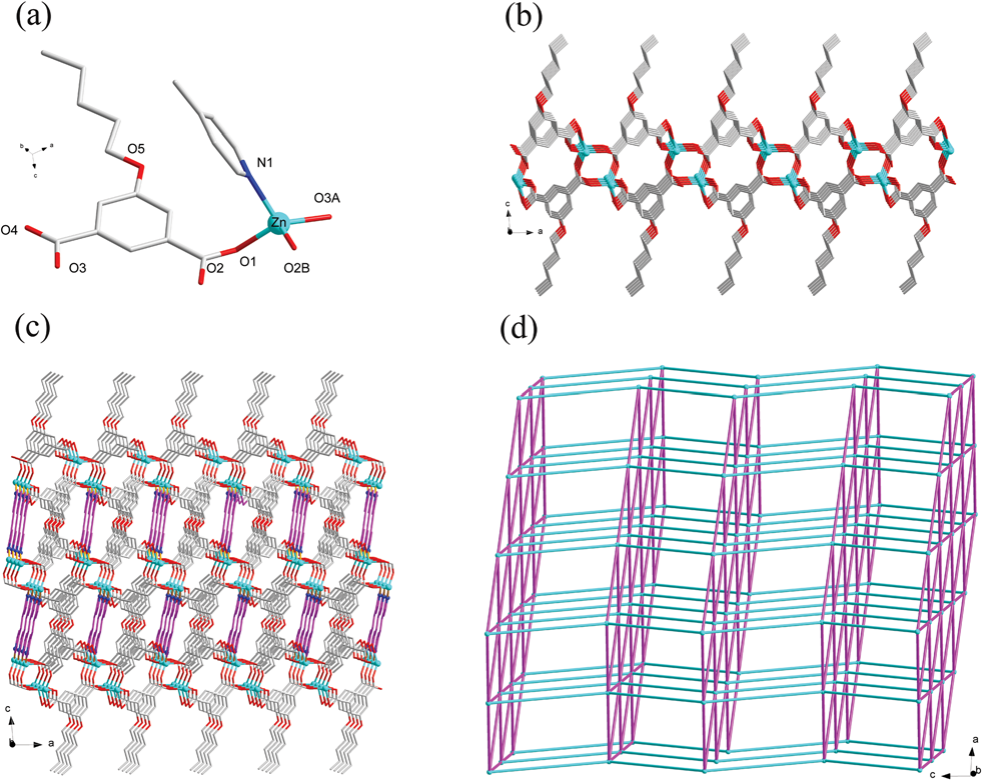
(a) Coordination environment of the Zn(ii) centers in 8 with the hydrogen atoms omitted for clarity (symmetric codes: (A) *x* + 1/2, *y* − 1/2, *z* and (B) −*x*, *y*, −*z* + 1/2). (b) The 2D layer constructed from ^*n*^PeOip ligands and Zn(ii) ions. (c) View of the 3D [Zn(^*n*^PeOip)(bpe)]_*n*_ coordination network of 8. (d) View of the topological net of 8.

Compound 8 crystallizes in the monoclinic system with the space group *C*2/*c*. The asymmetric unit of 8 is made up of one Zn(ii) ion, one ^*n*^PeOip and half of bpe. The four-coordinated Zn center is satisfied by three oxygen atoms from three different ^*n*^PeOip ligands and one nitrogen atom belonging to bpe, forming a distorted tetrahedral coordination [ZnN_1_O_3_] geometry (Fig. 7a). The Zn–O [1.9094(17) Å to 1.9831(17) Å] and Zn–N [2.0458(19) Å] distances are within typical values encountered in related Zn(ii) derivatives. In this structure, two adjacent Zn atoms of the same crystallography are combined together by two bis(monodentate) carboxylates to yield a bimetallic unit [Zn_2_(RCO_2_)_2_], in which the non-bonding distance of Zn_1_⋯Zn_1_ is 3.825 Å (Scheme 1(V) and Fig. S7†). The uniform bimetallic units are linked together by ^*n*^PeOip ligands to build a 2D layer (Fig. 7b). Each bpe ligand connects two Zn atoms from the adjacent 2D layers and extends the 2D layer substructures into a porous 3D framework (Fig. 7c). If the bimetallic units [Zn_2_(RCO_2_)_2_] are considered as nodes, ^*n*^PeOip ligands and bpe ligands as linkages, topological analysis reveals that compound 8 exhibits a 6-connected topological structure with a {3^3^·4^3^·5^5^·6^4^} point symbol (Fig. 7d).

### Effects of functionalized-groups of isophthalate and length of N-donor ligands on the structures of CPs

**Scheme 2:**
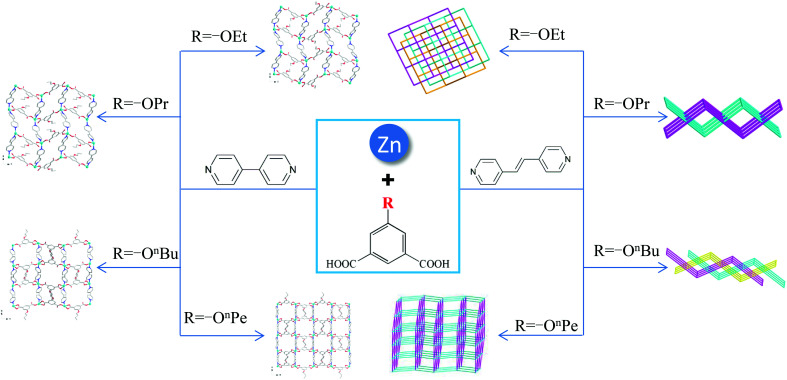
Summary of crystal structures in complexes 1–8.

It is well known that the structural diversities and properties of CPs are undoubtedly related to the functionalized groups of isophthalate derivative ligands, even though they are not involved in coordination with metal ions. As shown in Scheme 2, the reaction of EtOip, bpy, and Zn(ii) yielded a 2D layer (complex 1); similar structural framework were obtained when EtOip was replaced by PrOip (complex 2). When the PrOip ligand was replaced by ^*n*^BuOip complex 3 were obtained; similar structural networks were obtained when the ^*n*^PeOip ligand was used in the synthesis systems (complex 4). These results show that the EtO/PrO and ^*n*^BuO/^*n*^PeO functionalized-groups do not substantially affect the structural diversities of the resultant CPs. In comparison with bpy, bpe possessed distinct features (length), which would adjust the structural diversities and properties of the resultant CPs. The hydrothermal reaction of EtOip, bpe and Zn(ii) produced complex 5, with a structural framework is similar to that of MeOip;^35^ a slight variation of the functionalized-group from EtO to PrO resulted in the formation of a twofold interpenetrating 4-connected 2D framework (complex 6). A threefold interpenetrating 2D network (complex 7) is generated when the ^*n*^BuO group has inserts into the isophthalic group. Interestingly, when the functionalized-group ^*n*^BuO was replaced with ^*n*^PeO a non-interpenetrating 6-connected 3D framework (complex 8) were obtained. Evidently, the diverse structures of CPs 5–8 are seriously affected by the 5-functionalized groups of isophthalate derivative ligands. Although CPs 1 and 5, 2 and 6, 3 and 7, as well as 4 and 8 were prepared under similar reaction conditions except for the length of dipyridyl ligands used, distinct coordination frameworks were obtained. These results clearly demonstrated that different features of N-containing ligands (length) play a crucial role in determining the molecular structures of the resulting CPs.

### Thermal stability and PXRD

**Fig. 8:**
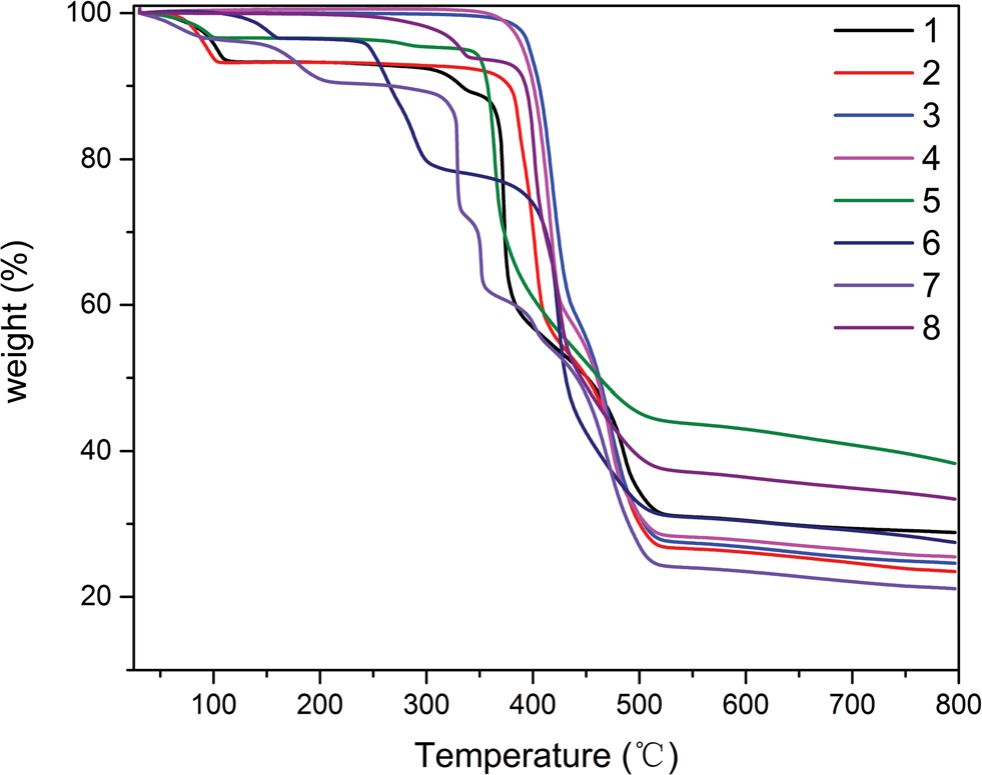
TGA curves of complexes 1–8.

The phase purities of 1–8 can be confirmed by powder X-ray diffraction (PXRD) patterns, which are in excellent agreement with the corresponding simulated ones (Fig. S9†), indicating the purity of the synthesized material and the as-simulated crystals. The thermal stability of compounds 1–8 was also studied by thermogravimetric analysis (TGA) in the 25–800 °C temperature range under N_2_ atmosphere. The obtained TGA curves for 1–8 are shown in Fig. 8. Compound 1 shows the loss of two crystallization water molecules (exptl, 7.9%; calcd, 8.1%) in the 50–115 °C range, followed by decomposition which begins at 300 °C. Compound 2 displays the removal of two crystallization water molecules in a temperature range from 60 °C to 105 °C (exptl, 7.2%; calcd, 7.5%), while a dehydrated sample then remains stable up to ∼350 °C. For compounds, 3, 4 and 8, which do not contain solvent molecules, TGA curves reveal that they are stable up to 350 °C, 342 °C and 260 °C, respectively, followed by decomposition upon further heating. The TGA curve of 5 shows a release of one H_2_O molecule between 50 °C and 113 °C (exptl, 3.5%; calcd, 3.9%), after which the compound was stable up to around 330 °C and then decomposed. In 6, a weight loss of approximately 3.6% corresponding to the loss of water molecules (calcd: 3.7%) in a temperature range of 100–162 °C, after which the compound was stable up to about 236 °C and then the compound decomposed gradually. The TGA profile of compound 7 showed several stages of weight loss with a 7.5% loss in a temperature range of 50–220 °C corresponding to the loss of half of the DMF molecules (calcd: 7.7%). Afterward the compound was stable up to around 300 °C and then the decomposed gradually over several steps.

### Solid state photoluminescence properties

**Fig. 9:**
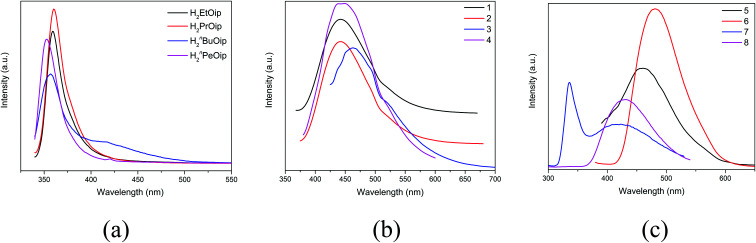
(a) The luminescence emission curves of free 5-functionalized isophthalate ligands (b) the luminescence emission curves of 1–4. (c) The luminescence emission curves of 5–8.

Taking into account the excellent luminescent properties of CPs with *d*^10^ metal centers,^6,7,67–70^ the luminescence of the free 5-functionalized isophthalate ligands and compounds 1–8 were investigated in the solid state at room temperature (Fig. 9). The free 5-functionalized isophthalate ligand displays photoluminescence with emission maxima at 360 nm (*λ*_ex_ = 345 nm) for H_2_EtOip, 360 nm (*λ*_ex_ = 347 nm) for H_2_PrOip, 357 nm (*λ*_ex_ = 334 nm) for H_2_^*n*^BuOip and 353 nm (*λ*_ex_ = 339 nm) for H_2_^*n*^PeOip, respectively, which may be assigned to the π* → n or π* → π transitions. Compounds 1–8 exhibit fluorescence bands at 442 nm (*λ*_ex_ = 348 nm) for 1, 442 nm (*λ*_ex_ = 353 nm) for 2, 463 nm (*λ*_ex_ = 397 nm) for 3, 442 nm (*λ*_ex_ = 325 nm) for 4, 456 nm (*λ*_ex_ = 361 nm) for 5, 482 nm (*λ*_ex_ = 357 nm) for 6, 336 nm and 433 nm (*λ*_ex_ = 272 nm) for 7, 433 nm (*λ*_ex_ = 272 nm) for 8, respectively. It is well known that Zn(ii) ions is difficult to oxidize or reduce due to its *d*^10^ configuration. Thus, the emissions of complexes 1–8 are neither metal-to-ligand charge transfer (MLCT) nor ligand-to-metal charge transfer (LMCT), which may be ascribed to a mixture character of intraligand and ligand-to-ligand charge transition (LLCT).

## Conclusions

In this work, eight Zn(ii) coordination polymers have been successfully synthesized under hydrothermal conditions by the reaction of 5-functionalized isophthalate acids and dipyridyl ligands with Zn(ii) salts. Although the 5-functionalized groups of isophthalate derivatives are not involved in coordination with Zn(ii) ions, their stericity and number of carbon atoms demonstrated significant effects on structural diversity, thermal stability and photoluminescent properties of the resultant CPs. These complexes show diverse structures varying from 2D to 3D frameworks. The variety of the structures indicates that 5-functionalized groups of isophthalate and the length of rigid dipyridyl ligands play a crucial important role in the self-assembly of CPs. Moreover, the thermal stabilities and photoluminescence properties were also studied. The results imply that these compounds are potential luminescent materials.

## Conflicts of interest

The authors declare no competing financial interest.

## Supplementary Material

RA-008-C7RA12874F-s001

RA-008-C7RA12874F-s002
