# Supplementary material for: Assembly of a series of zinc coordination polymers based on 5-functionalized isophthalic acids and dipyridyl
Source: RSC Adv. 2018 Feb 16;8(14):7428–37. doi: 10.1039/c7ra12874f (PMC9078387; doi:10.1039/c7ra12874f)
Supplement: RA-008-C7RA12874F-s001 [file RA-008-C7RA12874F-s001.pdf]

Electronic Supplementary Material (ESI) for RSC Advances.
This journal is © The Royal Society of Chemistry 2018

Electronic Supplementary Information (ESI)

Assembly of a series of zinc coordination polymers based on 5-functionalized Isophthalic Acids and dipyridyl

Tao Wang,† Rong-Rong Zhu,† Xiao-Feng Zhang, Tong Yan, Quan Wang, Jing Feng, Jie Zhou, Lin Du* and Qi-Hua Zhao*

Key Laboratory of Medicinal Chemistry for Natural Resource Education Ministry, School of Chemical Science and Technology · Pharmacy, Yunnan University, Kunming, 650091, PR China

* Corresponding author.

Email address: qhzhao@ynu.edu.cn; lindu@ynu.edu.cn

Table S1. Selected Bond Distances (Å) and Angles (deg) for Complexes **1-8**

		Complex **1**	
Zn1—O3^{i}	1.9475 (17)	O3^{i}—Zn1—O1	103.66 (8)
Zn1—O1	1.9554 (18)	O3^{i}—Zn1—N2^{ii}	134.60 (8)
Zn1—N2^{ii}	2.0396 (19)	O1—Zn1—N2^{ii}	108.52 (8)
Zn1—N1	2.079 (2)	O3^{i}—Zn1—N1	106.53 (8)
O3—Zn1^{iii}	1.9475 (17)	O1—Zn1—N1	98.10 (8)
N2—Zn1^{iv}	2.0396 (19)	N2^{ii}—Zn1—N1	99.76 (8)
		Complex **2**	
Zn1—O1	1.940 (2)	O1—Zn1—O3^{i}	103.93 (9)
Zn1—O3^{i}	1.9519 (18)	O1—Zn1—N2^{ii}	133.05 (9)
Zn1—N2^{ii}	2.030 (2)	O3^{i}—Zn1—N2^{ii}	109.24 (9)
Zn1—N1	2.077 (2)	O1—Zn1—N1	107.76 (9)
O3—Zn1^{iii}	1.9518 (18)	O3^{i}—Zn1—N1	97.15 (9)
N2—Zn1^{iv}	2.030 (2)	N2^{ii}—Zn1—N1	100.15 (9)
		Complex **3**	
Zn1—O1	1.935 (2)	O1—Zn1—N1	129.22 (10)
Zn1—O3^{i}	2.037 (2)	O3^{i}—Zn1—N1	104.61 (10)
Zn1—N1	2.067 (3)	O1—Zn1—N2^{ii}	100.96 (10)
Zn1—N2^{ii}	2.120 (3)	O3^{i}—Zn1—N2^{ii}	91.88 (10)
Zn1—O4^{i}	2.388 (2)	N1—Zn1—N2^{ii}	93.95 (11)
O4—Zn1^{iii}	2.388 (2)	O1—Zn1—O4^{i}	95.82 (9)
O3—Zn1^{iii}	2.037 (2)	O3^{i}—Zn1—O4^{i}	58.46 (9)
N2—Zn1^{iv}	2.120 (3)	N1—Zn1—O4^{i}	94.14 (10)
O1—Zn1—O3^{i}	122.84 (10)	N2^{ii}—Zn1—O4^{i}	150.33 (9)
		Complex **4**	
Zn1—N1	2.104 (4)	N1—Zn1—N1^{i}	94.1 (2)
Zn1—N1^{i}	2.104 (4)	N1—Zn1—O2	92.97 (15)
Zn1—O2	2.173 (4)	N1^{i}—Zn1—O2	150.89 (15)
Zn1—O2^{i}	2.173 (4)	N1—Zn1—O2^{i}	150.89 (15)
Zn1—O1	2.192 (4)	N1^{i}—Zn1—O2^{i}	92.97 (15)

Zn1—O1^{i}	2.192 (4)	O2—Zn1—O2^{i}	94.5 (2)
N1—Zn1—O1	101.89 (16)	N1^{i}—Zn1—O1^{i}	101.89 (15)
N1^{i}—Zn1—O1	91.19 (15)	O2—Zn1—O1^{i}	106.17 (15)
O2—Zn1—O1	59.71 (14)	O2^{i}—Zn1—O1^{i}	59.71 (14)
O2^{i}—Zn1—O1	106.17 (15)	O1—Zn1—O1^{i}	160.9 (2)
N1—Zn1—O1^{i}	91.19 (15)		
		Complex **5**	
Zn1—O1	1.979 (3)	O1—Zn1—O3^{i}	102.12 (12)
Zn1—O3^{i}	1.984 (3)	O1—Zn1—N2^{ii}	108.51 (13)
Zn1—N2^{ii}	2.030 (3)	O3^{i}—Zn1—N2^{ii}	111.52 (13)
Zn1—N1	2.043 (3)	O1—Zn1—N1	106.84 (13)
O3—Zn1^{iii}	1.984 (3)	O3^{i}—Zn1—N1	104.43 (13)
N2—Zn1^{iv}	2.030 (3)	N2^{ii}—Zn1—N1	121.63 (14)
		Complex **6**	
Zn1—O1	1.9264 (19)	O1—Zn1—N1	126.47 (9)
Zn1—O3^{i}	1.938 (2)	O3^{i}—Zn1—N1	96.08 (9)
Zn1—N1	2.048 (2)	O1—Zn1—N2	103.52 (10)
Zn1—N2	2.068 (3)	O3^{i}—Zn1—N2	104.93 (10)
O3—Zn1^{ii}	1.938 (2)	N1—Zn1—N2	105.37 (10)
O1—Zn1—O3^{i}	118.42 (9)		
		Complex **7**	
Zn1—O1	1.978 (2)	O1—Zn1—N2	107.55 (10)
Zn1—O4^{i}	1.980 (2)	O4^{i}—Zn1—N2	109.19 (11)
Zn1—N2	2.035 (2)	O1—Zn1—N1	107.61 (10)
Zn1—N1	2.037 (2)	O4^{i}—Zn1—N1	106.73 (11)
O4—Zn1^{ii}	1.980 (2)	N2—Zn1—N1	121.71 (11)
O1—Zn1—O4^{i}	102.39 (10)		
		Complex **8**	
Zn1—O3^{i}	1.9094 (17)	O3^{i}—Zn1—O1	121.25 (8)
Zn1—O1	1.9605 (16)	O3^{i}—Zn1—O2^{ii}	100.42 (8)

Zn1—O2^{ii}	1.9831 (17)	O1—Zn1—O2^{ii}	114.99 (7)
Zn1—N1	2.0458 (19)	O3^{i}—Zn1—N1	119.54 (8)
O2—Zn1^{ii}	1.9831 (17)	O1—Zn1—N1	98.95 (8)
O3—Zn1^{iii}	1.9095 (17)	O2^{ii}—Zn1—N1	100.54 (7)

Symmetry codes, **for 1:** (i) *x*-1/2, -*y*+3/2, *z*-1/2; (ii) -*x*-1/2, *y*+1/2, -*z*+1/2; (iii) *x*+1/2, -*y*+3/2, *z*+1/2; (iv) -*x*-1/2, *y*-1/2, -*z*+1/2**; for 2:** (i) *x*+1/2, -*y*+3/2, *z*+1/2; (ii) -*x*+1/2, *y*+1/2, -*z*+1/2; (iii) *x*-1/2, -*y*+3/2, *z*-1/2; (iv) -*x*+1/2, *y*-1/2, -*z*+1/2**; for 3:** (i) *x*, -*y*+1/2, *z*+1/2; (ii) -*x*+1, *y*-1/2, -*z*+1/2; (iii) *x*, -*y*+1/2, *z*-1/2; (iv) -*x*+1, *y*+1/2, -*z*+1/2**; for 4:** (i) -*x*+1/2, *y*, -*z*+1/2; (ii) *x*, -*y*+1, -*z*+1; (iii) -*x*+1, *y*, *z***; for 5**: (i) *x*+1, *y*, *z*+1; (ii) *x*-1, -*y*+1/2, *z*+1/2; (iii) *x*-1, *y*, *z*-1; (iv) *x*+1, -*y*+1/2, *z*-1/2; **for 6**: (i) *x*-1, *y*, *z*; (ii) *x*+1, *y*, *z*; (iii) -*x*+1, -*y*+3, -*z*+1; (iv) -*x*, -*y*+1, -*z*+1; **for 7:** (i) *x*+1, *y*+1, *z*; (ii) *x*-1, *y*-1, *z*; (iii) -*x*+1, -*y*+1, -*z*; (iv) -*x*+3, -*y*, -*z* **;** **for 8:** (i) *x*+1/2, *y*-1/2, *z*; (ii) -*x*, *y*, -*z*+1/2; (iii) *x*-1/2, *y*+1/2, *z*; (iv) -*x*, -*y*+2, -*z*.

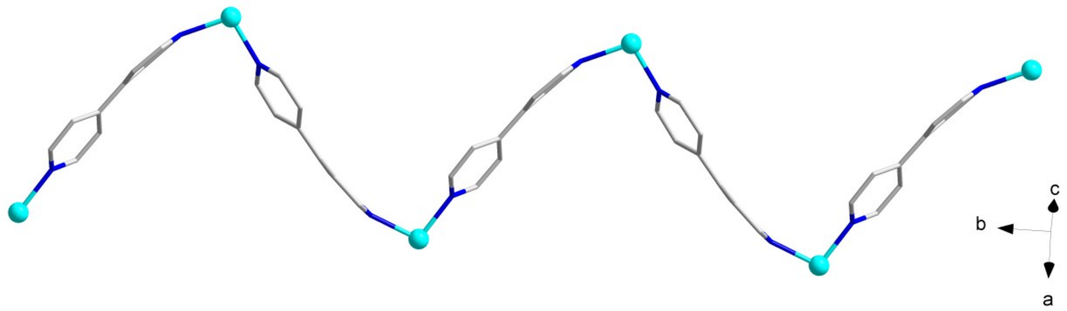

Fig. S1. View of the 1D zigzag chains constructed by bpy and Zn(II) in **1** and **2**.

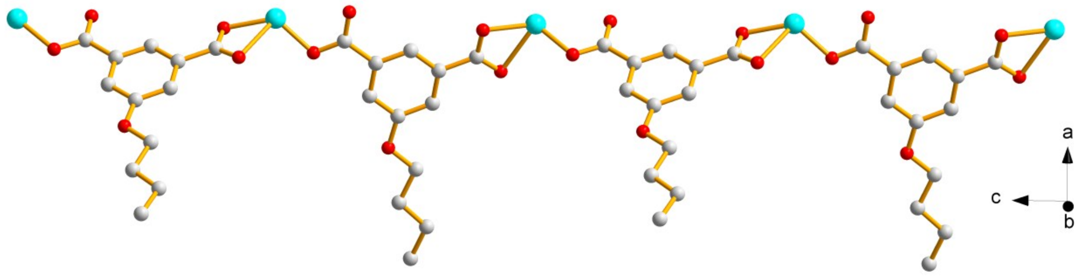

Fig. S2. View of the 1-D chain constructed by ^{n}BuOip and Zn(II) in **3**.

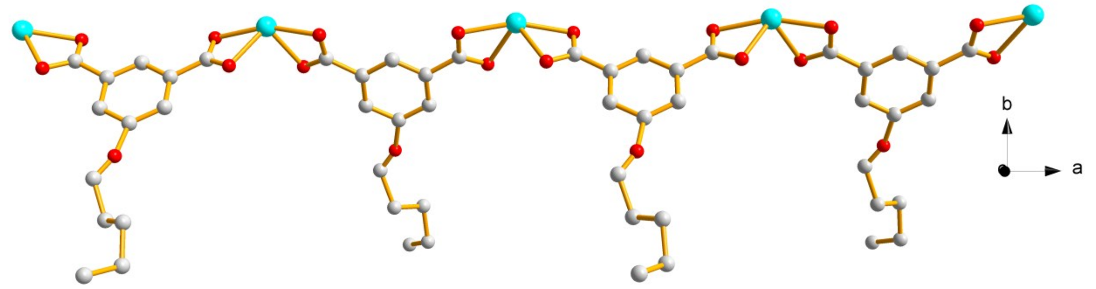

Fig. S3. View of the 1-D chain constructed by ^{n}PeOip and Zn(II) in **4**.

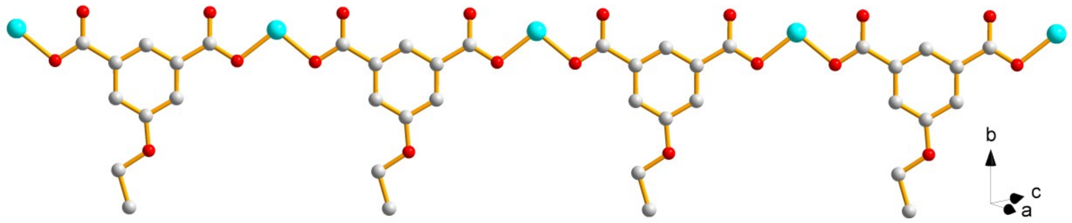

Fig. S4. View of the 1-D chain constructed by EtOip and Zn(II) in **5**.

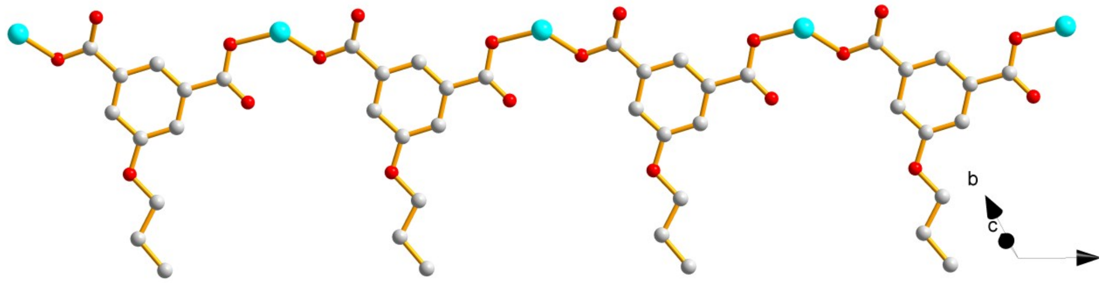

Fig. S5. View of the 1-D chain constructed by PrOip and Zn(II) in **6**.

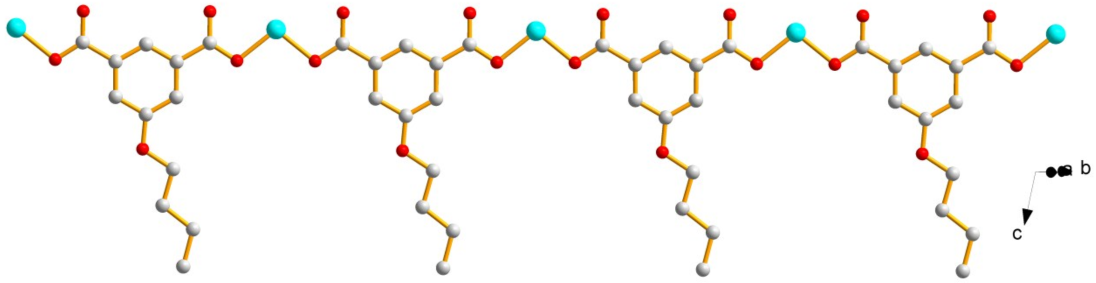

Fig. S6. View of the 1-D chain constructed by ^{n}BuOip and Zn(II) in **7**.

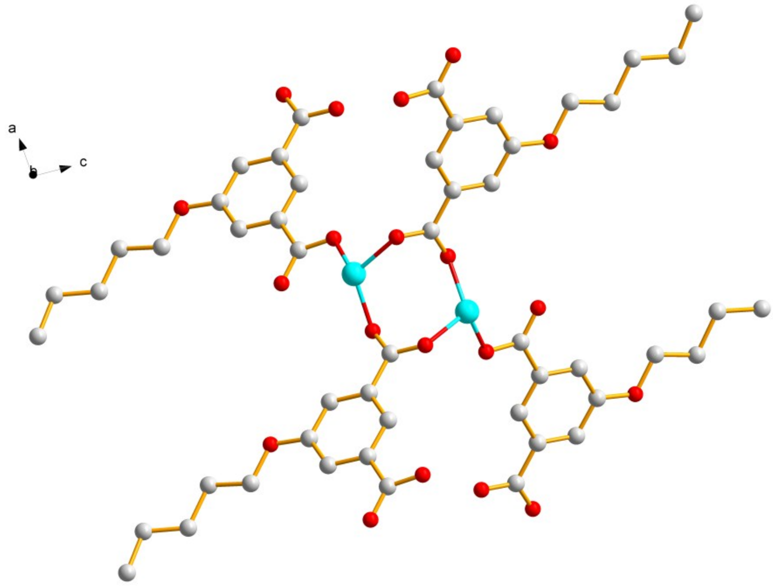

Fig. S7. View of a dinuclear $[Zn_2(^{n}PeOip)_2]$ unit in **8**.

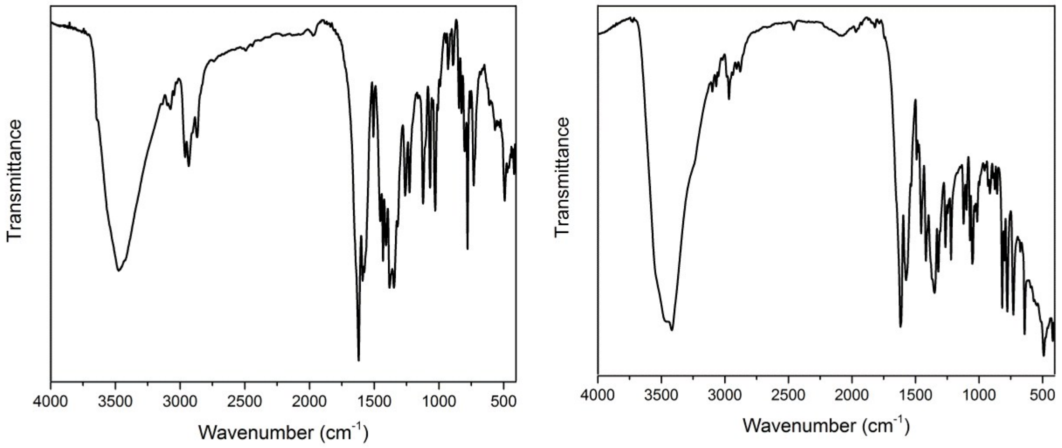

(a) (b)

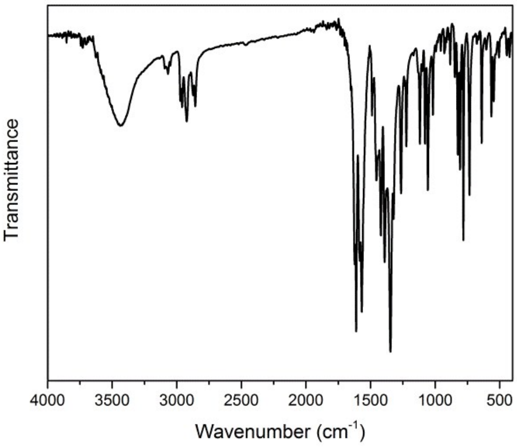

(c)

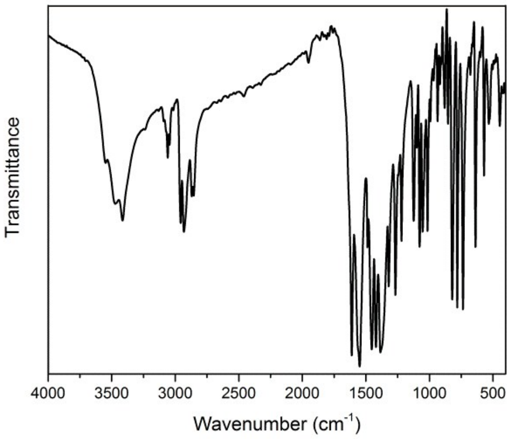

(d)

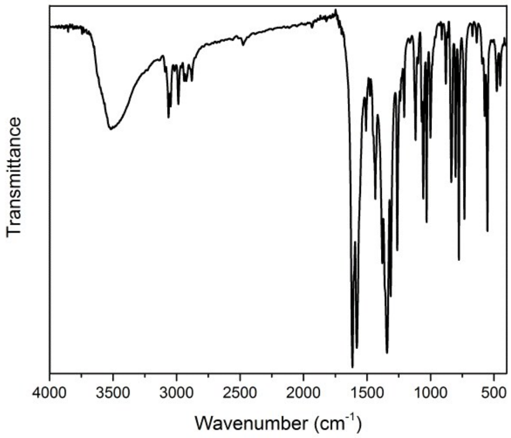

(e)

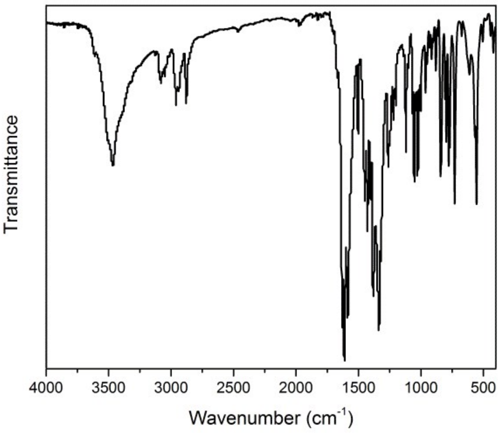

(f)

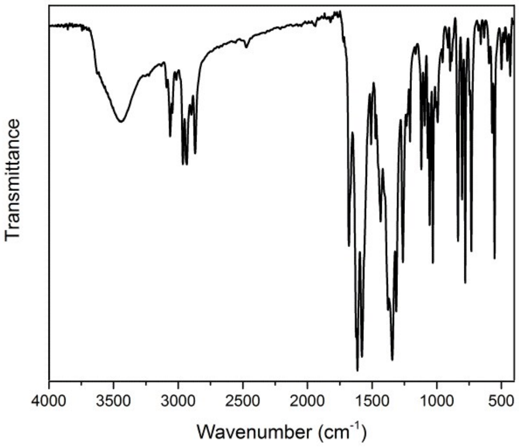

(g)

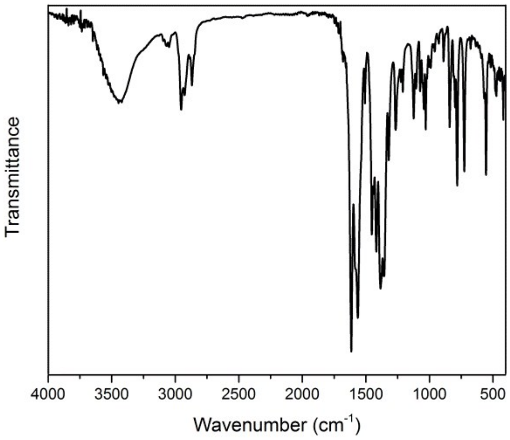

(h)

Fig. S8. FT-IR spectrum for **1** (a); **2** (b); **3** (c); **4** (d); **5** (e); **6** (f); **7** (g) and **8** (h).

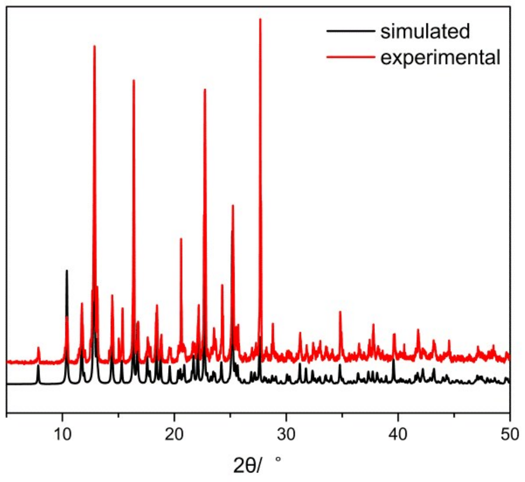
simulated
experimental
10 20 30 40 50
2θ/ °

(a)

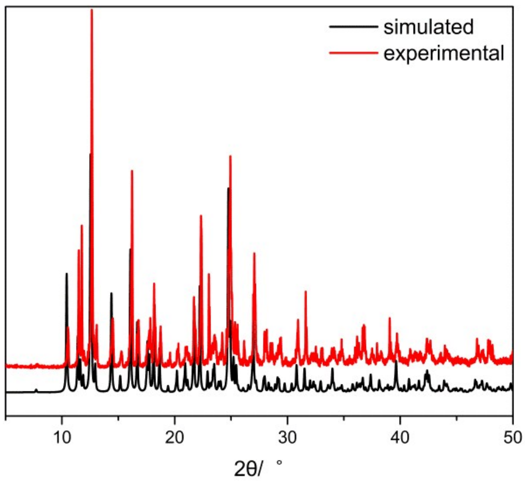
simulated
experimental
10 20 30 40 50
2θ/ °

(b)

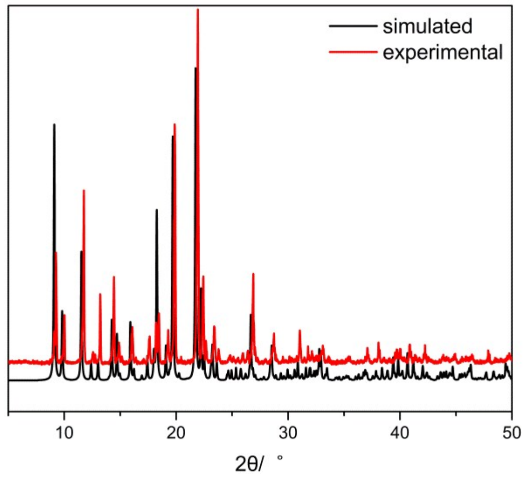
simulated
experimental
10 20 30 40 50
2θ/ °

(c)

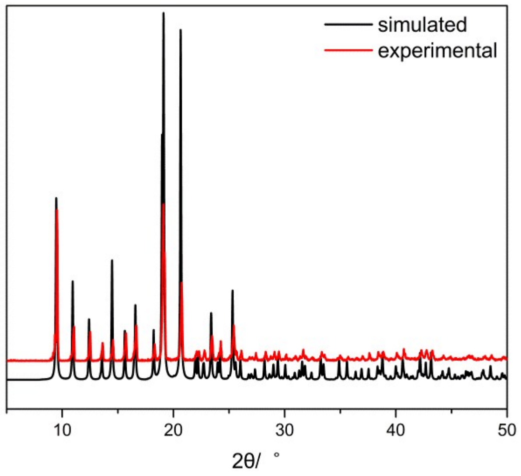
simulated
experimental
10 20 30 40 50
2θ/ °

(d)

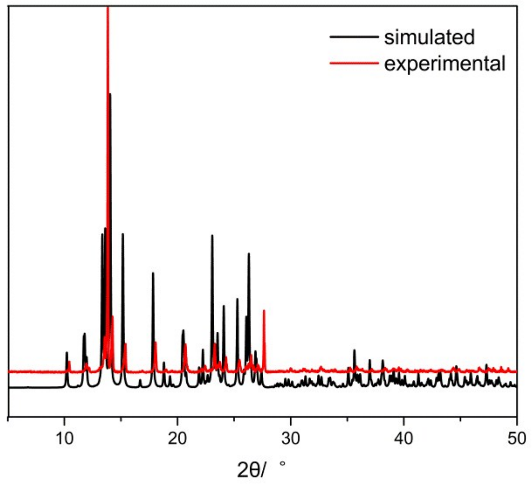
simulated
experimental
10 20 30 40 50
2θ/ °

(e)

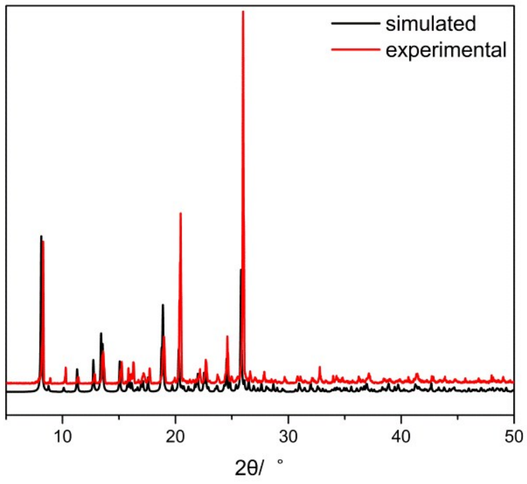
simulated
experimental
10 20 30 40 50
2θ/ °

(f)

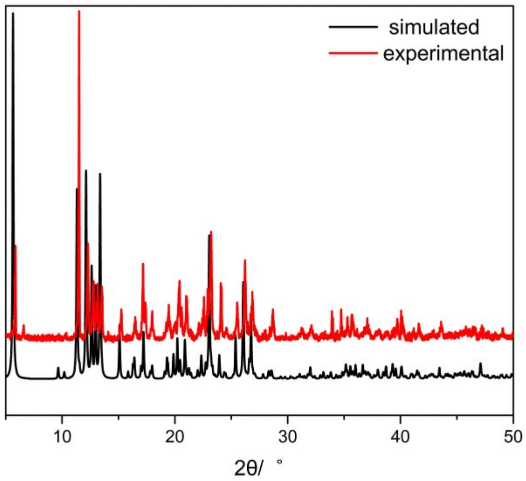

(g)

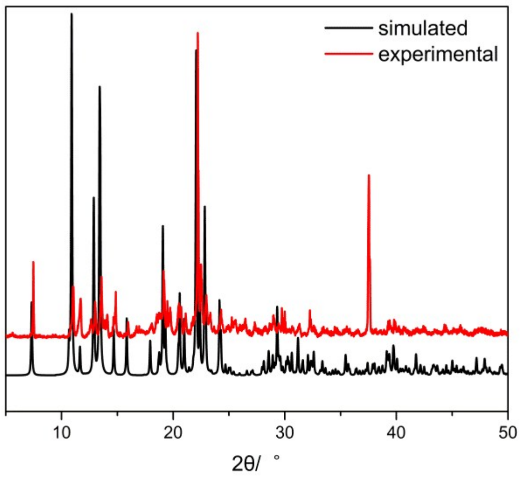

(h)

Fig. S9. Powder XRD patterns for **1** (a); **2** (b); **3** (c); **4** (d); **5** (e); **6** (f); **7** (g) and **8** (h). Black: simulated from single crystal analysis and experimental; Red: as synthesized.
